# α_1A_-Adrenergic Receptor Induces Activation of Extracellular Signal-Regulated Kinase 1/2 through Endocytic Pathway

**DOI:** 10.1371/journal.pone.0021520

**Published:** 2011-06-28

**Authors:** Fei Liu, Kangmin He, Xinxing Yang, Ning Xu, Zhangyi Liang, Ming Xu, Xinsheng Zhao, Qide Han, Youyi Zhang

**Affiliations:** 1 Institute of Vascular Medicine, Peking University Third Hospital, and Key Laboratory of Molecular Cardiovascular Sciences, Ministry of Education, Beijing, China; 2 Beijing National Laboratory for Molecular Sciences, State Key Laboratory for Structural Chemistry of Unstable and Stable Species, Department of Chemical Biology, College of Chemistry and Molecular Engineering, Biodynamic Optical Imaging Center, Peking University, Beijing, China; Cornell University, United States of America

## Abstract

G protein-coupled receptors (GPCRs) activate mitogen-activated protein kinases through a number of distinct pathways in cells. Increasing evidence has suggested that endosomal signaling has an important role in receptor signal transduction. Here we investigated the involvement of endocytosis in α_1A_-adrenergic receptor (α_1A_-AR)-induced activation of extracellular signal-regulated kinase 1/2 (ERK1/2). Agonist-mediated endocytic traffic of α_1A_-AR was assessed by real-time imaging of living, stably transfected human embryonic kidney 293A cells (HEK-293A). α_1A_-AR was internalized dynamically in cells with agonist stimulation, and actin filaments regulated the initial trafficking of α_1A_-AR. α_1A_-AR-induced activation of ERK1/2 but not p38 MAPK was sensitive to disruption of endocytosis, as demonstrated by 4°C chilling, dynamin mutation and treatment with cytochalasin D (actin depolymerizing agent). Activation of protein kinase C (PKC) and C-Raf by α_1A_-AR was not affected by 4°C chilling or cytochalasin D treatment. U73122 (a phospholipase C [PLC] inhibitor) and Ro 31–8220 (a PKC inhibitor) inhibited α_1B_-AR- but not α_1A_-AR-induced ERK1/2 activation. These data suggest that the endocytic pathway is involved in α_1A_-AR-induced ERK1/2 activation, which is independent of G_q_/PLC/PKC signaling.

## Introduction

α_1A_-Adrenergic receptor (α_1A_-AR) is one of 3 members of the α_1_-AR subfamily (α_1A_, α_1B_, and α_1D_) of G protein-coupled receptors (GPCRs) [1]–[3]. α_1A_-AR plays a key role in physiological effects such as contraction of vascular and cardiac muscle, contraction of the spleen, liver glycogenesis, or melatonin secretion in the pineal gland [2]–[5]. Mice with cardiac-restricted overexpression of the wild-type α_1B_-AR that were treated with α_1_-AR agonist (phenylephrine [PE]) exhibited poor survival, markedly exaggerated cardiac hypertrophy, myocardial fibrosis, and suppressed left ventricular function [6]. In contrast, animals with α_1A_-AR overexpression showed improved survival and even abrogated cardiac remodeling in response to thoracic aorta constriction-induced pressure overload or myocardial infarction [7], [8]. The activation of extracellular signal-regulated kinase (ERK), a regulator of myocyte survival, is critical in mediating α_1_-AR survival signaling in cardiac myocytes [9]–[11]. Recent studies of selective inactivation of α_1_-ARs indicate that the activation of ERK1/2 induced by α_1A_-AR is critical for cardiomyocyte survival. Reconstitution of α_1A_-AR but not α_1B_-AR induced ERK1/2 activation and rescued α1ABKO myocytes from cell death induced by norepinephrine, doxorubicin, and H_2_O_2_ [12]. The observation that α_1A_-AR specifically restored ERK1/2 activation in α1ABKO myocytes suggests that α_1A_-AR and α_1B_-AR activate ERK1/2 through differential mechanisms. However, studies to date have not consistently identified major differences in immediate signaling responses initiated by α_1A_-AR and α_1B_-AR.

α_1A_-AR is regulated by many mechanisms, including phosphorylation, protein-protein interaction, protein traffic, and transcription [13]. After stimulation by their ligands, α_1_-ARs activate intracellular effectors, including phospholipase C β (PLCβ), inositol trisphosphate, protein kinase C (PKC), mitogen-activated protein kinase (MAPK) and calcium signals, often through a heterotrimeric G protein-dependent manner [14], [15]. An α_1A_-AR variant, which was unable to couple to G_q_, could also induce calcium influx when coactivated by β_2_-AR [16]. Thus, α_1A_-AR, even though uncoupled from G_q_, may remain competent for induction of signaling events through yet unknown pathways.

Increasing evidence has shown the existence of receptor signaling from the endocytic process. For instance, activation of ERK1/2 via epidermal growth factor receptor (EGFR) and β_2_-AR were suppressed in cells transfected with dynamin-mutant K44A (Dyn-K44A), which is defective in GTPase activity [17], [18]. Signaling from GPCR inside the cell is persistent and appears to trigger specific downstream effects [19]. Visualizing and tracking receptors stimulated by agonists in living cells contributes to understanding the molecular mechanisms of receptor signaling [20]. However, the association of α_1A_-AR endocytic trafficking and activation of MAPKs is still unknown.

We aimed to investigate whether an endocytic process is involved in ERK1/2 activation induced by α_1A_-AR. By real-time tracking, we found a time-dependent dynamic pattern of α_1A_-AR endocytosis with stimulation and the involvement of the cytoskeleton, especially actin-filaments, in this process. This relationship was further examined by colocalization of α_1A_-AR with reorganized cytoskeletons. We provide evidence for an involvement of endocytosis in α_1A_-AR-induced activation of ERK1/2, which differs from that of α_1B_-AR via a G_q_/PLC/PKC pathway.

## Materials and Methods

### Materials

Cytochalasin D, nocodazole, PE and U73122 were from Sigma (St. Louis, MO). Ro 31–8220, prazosin and phorbol 12-myristate, 13-acetate (PMA) were from Calbiochem (La Jolla, CA). Phospho-p38 MAPK (Thr180/Tyr182), -p42/44 MAPK (Thr202/Tyr204), -PKC (pan) (Ser660), and -C-Raf (Ser338) antibodies were from Cell Signaling Technology (Beverly, Mass). Antibodies against ERK1/2, PKC (pan), C-Raf, p38, FLAG-tag and HA-tag were from Santa Cruz Biotechnology (Santa Cruz, CA). Horseradish peroxidase (HRP)-conjugated goat anti-mouse and anti-rabbit antibodies were from Beijing Zhongshan Golden Bridge Biotechnology. Alexa 488-conjugated WGA, Alexa 555 and 633 IgG and Alexa 488-conjugated phalloidin were from Invitrogen. All other chemicals were of analytical grade.

### Cell culture, plasmids and transfection

HEK-293A cell lines were obtained from Invitrogen. Receptor constructs and HEK-293A cells stably transfected with α_1A_-AR or α_1B_-AR were described previously [21]. Dyn-K44A was a gift from Ming Zhao (La Jolla Institute for Molecular Medicine, San Diego, CA). Amphiphysin I construct was a gift from Pietro De Camilli (Yale University School of Medicine, New Haven, CT). Transfection involved use of Lipofectamine 2000 (Invitrogen) according to the manufacturer's instructions.

### Membrane receptor labeling and tracking

FLAG-tagged receptors were labeled with anti-FLAG monoclonal antibody (12.5 µg/ml) for 10 min and then Alexa FLour® 555 goat anti-mouse IgG (Invitrogen) (3.75 µg/ml) for 10 min as described previously [21], [22]. Before fluorescence experiments, cells were washed 3 times in phosphate buffered saline (PBS) buffer (pH 7.4; 37°C). Live imaging involved use of a wide-field fluorescence microscope equipped with a 100×/1.40NA Plan Apochromat objective (Olympus, Japan) and a 14-bit, back-illuminated, electron-multiplying charge-coupled device camera (Andor iXon DU-897 BV). The microscope was also equipped with a cell incubation system (INU-ZIL-F1, TOKAI HIT), which ensured live-cell imaging at 37°C in 5% CO_2_. Fluorescence was excited at 532-nm by an argon laser (Melles Griot, Carlsbad, CA). Movies were acquired at a frame rate of 20 Hz by use of MetaMorph software (Molecular Devices). Trajectories from cells observed under the given labeling procedure were plotted and resolved as described previously [21].

### Fluorescence microscopy

After drug treatment, cells were fixed for 15 min in 4% paraformaldehyde in PBS and permeabilized with 0.2% Triton X-100. After washes with PBS, cells were incubated for 25 min with TRITC-labeled phalloidin (Sigma). The samples were viewed under a laser scanning confocal microscope (TCS SP2, Leica Microsystems) with a Plan-Apo 63×/1.32 oil immersion objective (Leica Microsystems); images were collected by use of Leica TCS SP2 v2.611537. The 488- and 532-nm laser beam was focused by a Leica Apochromat with <200 lW power irradiation. The pinhole size was 1 airy unit.

### Western blot analysis

Protein expression was examined by western blot analysis as previously described [23]. Briefly, samples were separated by 10% SDS-PAGE and transferred to nitrocellulose membranes. After being blocked, blots were probed with the appropriate primary antibodies overnight at 4°C or for 2 h at room temperature, then washed and incubated with HRP-conjugated secondary antibody. Bands were visualized by use of a super-western sensitivity chemiluminescence detection system (Pierce). Autoradiographs were quantitated by densitometry (Science Imaging System, Bio-Rad).

## Results

### Tracking α_1A_-AR stimulated by agonist in single living cells in real time

**Figure 1:**
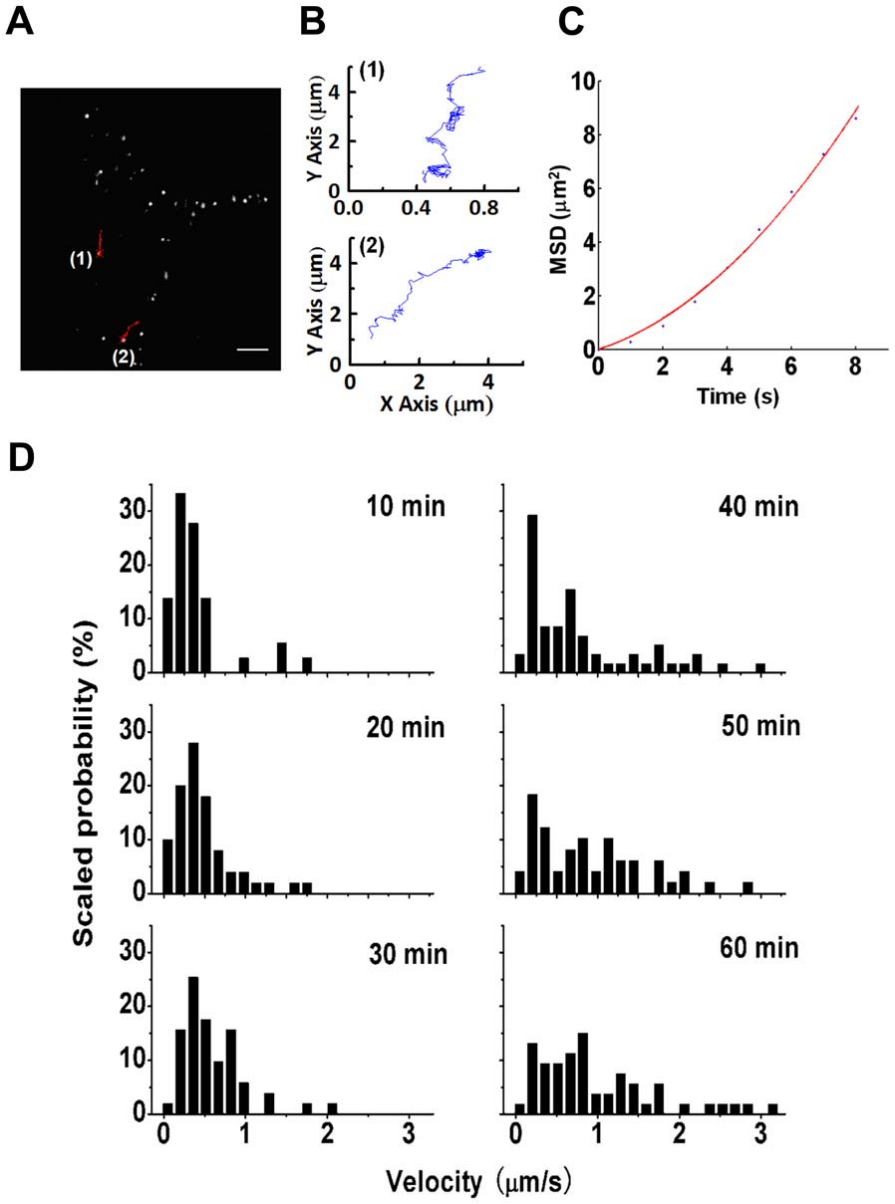
Tracking α_1A_-AR in response to agonist stimulation. (A) α_1A_-ARs were detected with anti-FLAG antibody and Alexa-555 IgG in live HEK-293A–α_1A_-AR cells at 37°C. Images were captured after 30-min stimulation with 10 µM phenylephrine (PE). Two sample trajectories of α_1A_-AR particles are shown with red lines (1 and 2). Bar: 10 µm. (B) The trajectories in (A) were plotted (1 and 2, respectively). (C) The plot of the mean square displacement (<r^2^>) against time (t) to the trajectory in Fig. B(2). The red line is a fit by <r^2^> = 4Dt+(vt)^2^. Directed movement was confirmed by the superlinear MSD-Δt plots. (D) Velocities of directional movements of α_1A_-AR resolved from tracked trajectories at various times after 10 µM PE stimulation plotted in probability histograms. (n = 36, 61, 51, 58, 49 and 53 trajectories in separated cells, respectively).

We studied the dynamic properties and mechanisms of receptor transport in HEK-293A cells stably transfected with a FLAG-tagged α_1A_-AR construct. α_1A_-AR was detected on the surface of living HEK-293A–α_1A_-AR cells by use of a monoclonal primary antibody and Alexa-555 IgG (Fig. 1A). After incubation with PE, an α_1_-AR agonist, some of the α_1A_-AR particles trafficked inward in the cells. From recorded movies, we tracked the trajectories of trafficking α_1A_-AR particles. Figure 1B shows 2 sample trajectories of α_1A_-AR particles (as marked in Fig. 1A) with directed movement within 8 sec on PE stimulation. To quantify the velocities of α_1A_-AR movements, we plotted the mean square displacement (MSD) *versus* time (Fig. 1C), which also showed the directional movement of these particles.

We then resolved the velocities at different time after PE stimulation [21]. Figure 1D shows the time-dependent velocity distribution of endocytic α_1A_-AR with PE stimulation during 1 hour (10-min intervals). At the early stage of the activation (first 30 min), the receptor mainly moved at a low velocity at a peak of about 0.3 µm/s. After stimulation for 40 to 60 min, movements became much faster, with high velocity trajectories increased gradually. The main peak of the highest velocity was about 0.8 µm/s. Thus, in general, the active movement of α_1A_-AR vesicles was slower at the early phase of endocytosis and faster at the later phase.

### Actin filament-mediated endocytosis of α_1A_-AR

**Figure 2:**
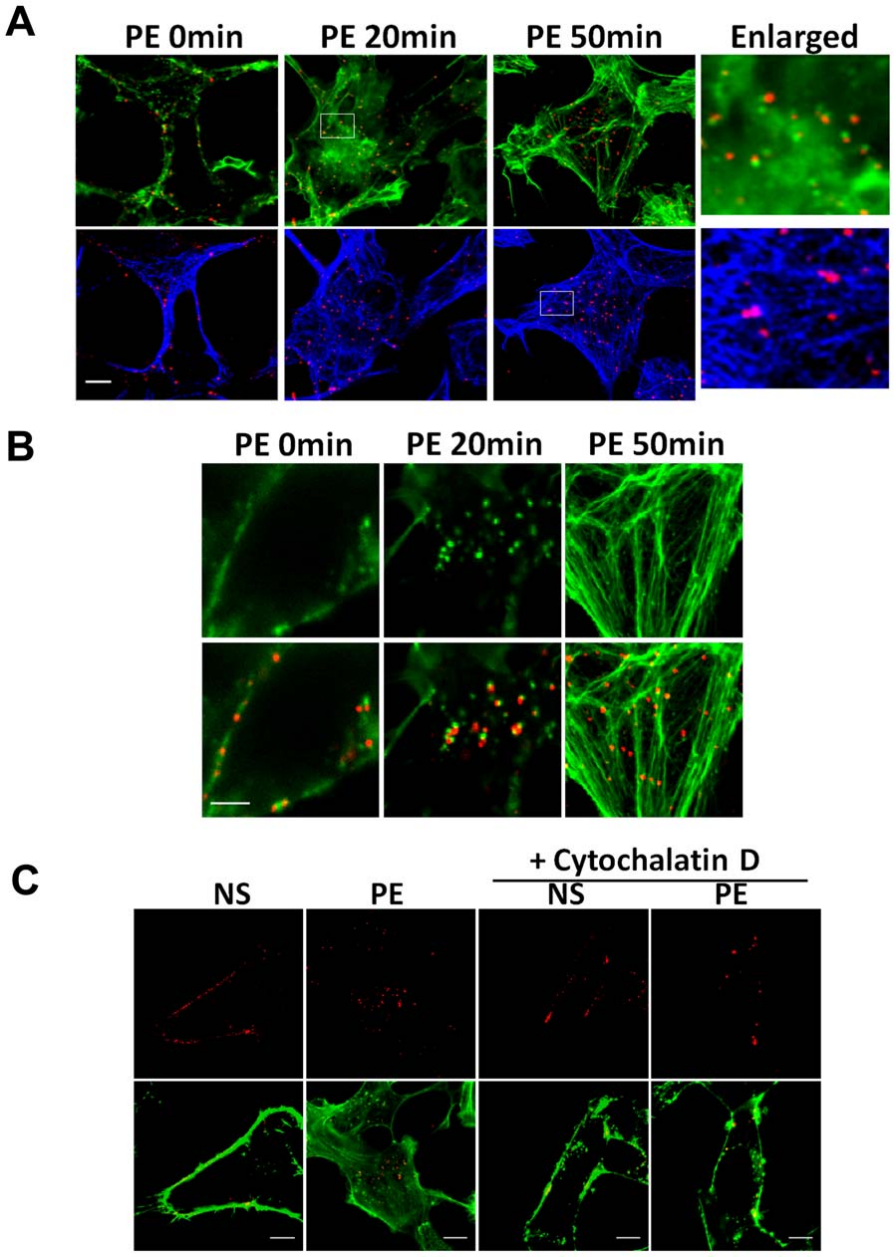
α_1A_-AR endocytosis is regulated by cytoskeleton. (A) Colocalization of α_1A_-AR with F-actin and microtubules after agonist stimulation. Cells were stimulated with 10 µM PE for 20 or 50 min. Untreated cells were used as control. α_1A_-AR was labeled with anti-FLAG antibodies and Alexa 555 IgG (*red*). F-actin was labeled with Alexa 488-conjugated phalloidin (*green*). Microtubules were labeled with antibodies and Alexa 633 IgG (*blue*). Last column: 5× magnification of selected boxed regions. Bar: 10 µm. (B) High-resolution imaging of colocalization of α_1A_-AR with reorganized actin after stimulation. Cells were treated with agonist for 20 or 50 min, and then labeled with antibodies against α_1A_-AR and Alexa 488-conjugated phalloidin against F-actin (red) (bottom row). Bar: 5 µm. (C) Inhibition of α_1A_-AR endocytosis by Cytochalasin D. HEK-293A–α_1A_-AR cells were pre-incubated with cytochalasin-D (Cyto-D; 5 µM, 5 min), then stimulated with 10 µM PE for 20 min. F-actin was stained by Alexa 488-conjugated Phalloidin (*green*), α_1A_-ARs were detected with anti-FLAG antibody and Alexa-555 IgG (*red*). NS: no stimulation. Bar: 10 µm.

We used confocal microscopy to determine the association of endocytic receptors with cytoskeleton, actin and microtubules, respectively. α_1A_-AR vesicles largely colocalized with F-actin after 20-min PE stimulation (Fig. 2A). With 50-min PE stimulation, some of the α_1A_-AR vesicles colocalized with microtubules. With higher resolution imaging, we observed a more relevant relation between reorganized actin and α_1A_-AR; at 20 min after PE stimulation, small actin patches and tails appeared in the cells (Fig. 2B). Most of the actin patches showed colocalization of a α_1A_-AR vesicle. Actin may use α_1A_-AR-associated actin patches as polymerization sites, as was reported for virus internalization [24]. The changes were transient, and after 50-min stimulation, most of actin patches and tails disappeared. And α_1A_-AR vesicles became located on the filamentous actin. Thus, PE-induced α_1A_-AR endocytic trafficking in the early phase depends on F-actin.

To justify the role of F-actin in regulation of α_1A_-AR endocytosis, cytochalasin D was used before PE stimulation to inhibit the actin polymerization. Incubated for 5 min with 5 µM cytochalasin D, α_1A_-AR congregated on membrane even after PE stimulation (Fig. 2C). It provides further evidence that α_1A_-AR endocytosis is regulated by actin filaments.

### Receptor endocytosis is required for ERK1/2 activation induced by α_1A_-AR

**Figure 3:**
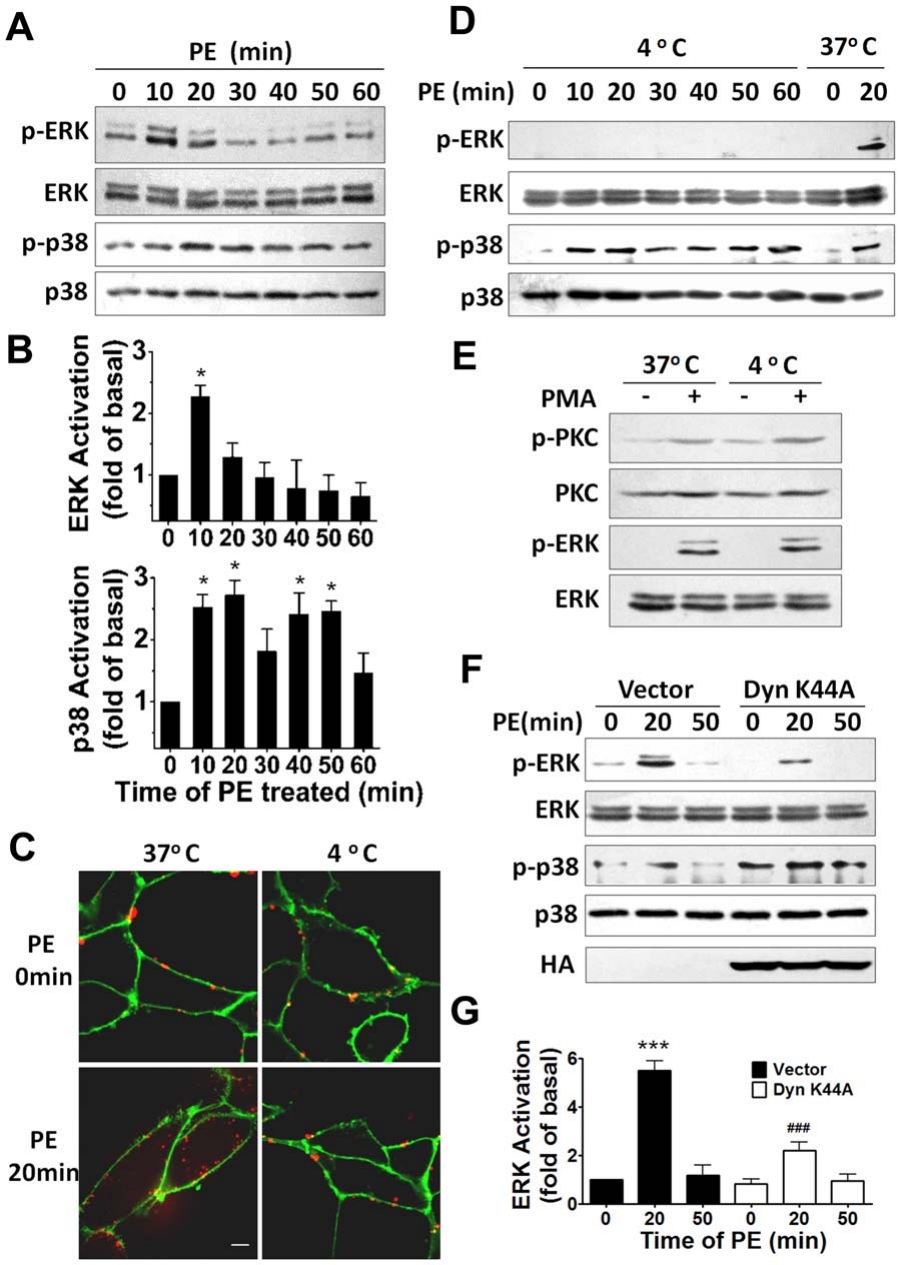
Receptor endocytosis is involved in ERK1/2 activation by α_1A_-AR stimulation. (A) Representative western blot showing activation of ERK1/2 and p38 after 10-µM PE treatment for the indicated times. (B) Relative ERK1/2 and p38 activeity after PE stimulation are shown. Data are means±SEM of results obtained in three independent experiments. Statistical significance of the difference was assessed using one-way ANOVA analysis. *, *p*<0.05 versus 0 min. (C) Effect of 4°C chilling on agonist-induced α_1A_-AR endocytosis. Cells were incubated in 4°C or 37°C for 30 min then stimulated with 10 µM PE for 20 min. α_1A_-ARs were detected with anti-FLAG antibody and Alexa-555 IgG (*red*), and plasma membrane was labeled with Alexa 488-conjugated WGA (*green*). (D) Western blot analysis of ERK1/2 and p38 phosphorylation with PE stimulation for the indicated times at 4°C. Stimulation at 37°C is a control. (E) Phorbol 12-myristate, 13-acetate (PMA)-induced activation of protein kinase C (PKC) and ERK1/2 at 4°C. Cells were incubated in 4°C or 37°C for 30 min then stimulated with 0.1 µM PMA for 20 min. (F) Dynamin mutation inhibits the activation of ERK1/2 by α_1A_-AR. HEK-293A–α_1A_-AR cells were cultured and infected with Dyn-K44A or vectors. After 40 h, cells were treated for 20 min with 10 µM PE. Protein expression of phospho-ERK1/2 and total ERK1/2 and p38 were measured. Expression of Dyn-K44A (HA-tagged) was identified with blotting of HA-tag. (G) Quantification of relative ERK1/2 activation corresponding to (F) was performed by densitometric analysis. Data are means±SEM of results obtained in three independent experiments. Statistical significance of the difference was assessed using one-way ANOVA analysis. ***, p<0.001 versus Vector 0 min. ^###^, *p*<0.001 versus Vector 20 min.

To test whether endocytosis is involved in the α_1A_-AR induced signaling, we first examined the activation of ERK1/2 and p38 MAPK with PE stimulation. ERK1/2 and p38 MAPK phosphorylation significantly increased at 10 and 20 min after PE treatment and then decreased to the basal level (Fig. 3A,B). PE also caused a secondary increase of p38 MAPK phosphorylation after 50-min treatment. We then used 4°C incubation to inhibit α_1A_-AR endocytosis [25], [26]. α_1A_-AR remained on the membrane after PE stimulation at 4°C (Fig. 3C). α_1A_-AR endocytosis was markedly inhibited at 4°C as compared with at 37°C. 4°C chilling almost completely abrogated the α_1A_-AR-induced ERK1/2 activation, whereas activation of p38 was not modified (Fig. 3D). To ensure that the ERK1/2 was not defective in phosphorylation at 4°C incubation, we measured PMA-induced activation of ERK1/2 in 4°C. PMA activated both PKC and ERK1/2 at 4°C and at 37°C (Fig. 3E), which suggests that the failure to activate ERK1/2 by α_1A_-AR at 4°C was not due to a defect in ERK1/2 signaling but rather to a defect in receptor endocytosis.

To further define the role of endocytosis in α_1A_-AR induced signaling, we tested the effect of the mutant Dyn-K44A, used to induce trafficking defects of receptors in many cells [27], [28], on ERK1/2 and p38 activation. Overexpression of HA-tagged Dyn-K44A in HEK-293A–α_1A_-AR cells suppressed the activation of ERK1/2 but not p38 (Fig. 3F, 3G). Therefore, endocytosis is required for α_1A_-AR-induced ERK1/2 but not p38 MAPK activation.

### Actin organization is involved in ERK1/2 activation induced by α_1A_-AR

**Figure 4:**
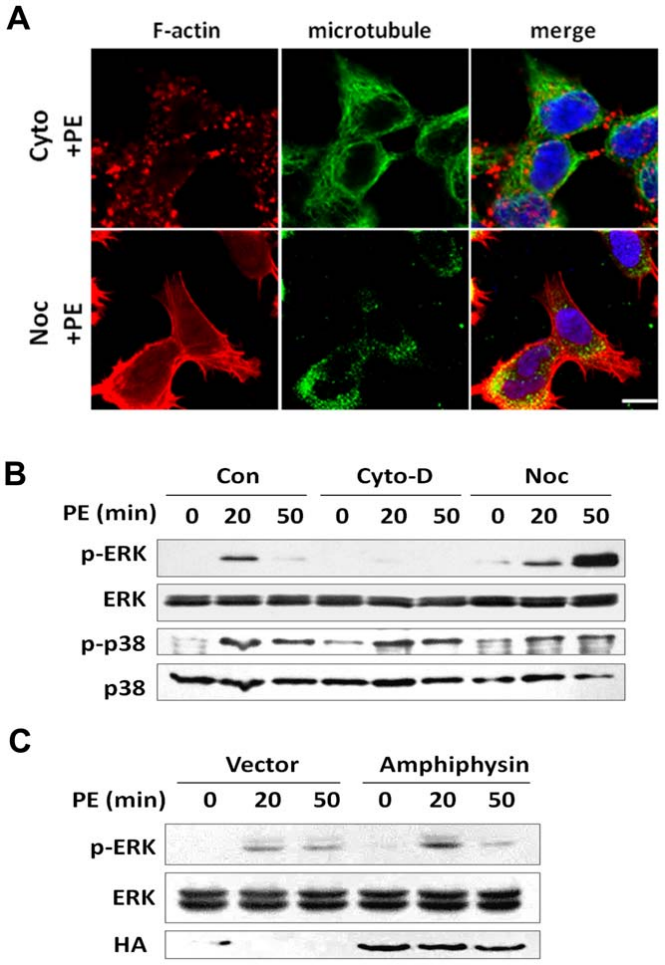
Actin organization is involved in ERK1/2 activation induced by α_1A_-AR. (A) Effect of actin and microtubule-disrupting drugs on cytoskeleton organization after agonist stimulation. HEK-293A–α_1A_-AR cells were pre-incubated with cytochalasin-D (Cyto-D; 5 µM, 5 min) or nocodazole (noc; 20 µM, 20 min) at 37°C, then stimulated with 10 µM PE for 20 min. F-actin was stained by TRITC-conjugated Phalloidin (*red*), and microtubules were stained with anti-α-tubulin antibody and Alexa-488 IgG (*green*). Nuclei were stained with Hochest-33342 (*blue*). Bar: 10 µm. (B) Effect of cytoskeleton disrupton on the activation of ERK1/2 and p38 after α_1A_-AR stimulation. HEK-293A–α_1A_-AR cells were pre-incubated with Cyto-D (5 µM, 5 min) or nocodazole (20 µM, 20 min) in 37°C, then treated with 10 µM PE for 20 and 50 min. Cell lysates were immunoblotted for the phosphrylation of ERK1/2 and p38. (C) Overexpression of amphiphysin enhanced the activation of ERK1/2 after agonist stimulation. HEK-293A–α_1A_-AR cells were transfected with amphiphysin plasmid, then treated with 10 µM PE for 20 and 50 min. Cell lysates were immunoblotted for analysis of phospho and total ERK1/2.

Actin filaments play a pivotal role in α_1A_-AR trafficking, especially in the early phase of endocytosis (Fig. 1, 2). Therefore, we tested the contribution of actin polymerization to α_1A_-AR-induced activation of ERK1/2. We pretreated cells with cytochalasin D or nocodazole before PE stimulation to disrupt to disrupt organizing actin or microtubules, respectively. In cells treated for 5 min with 5 µM cytochalasin D, filamentous form of actin was depolymerized and was replaced by aggregated actin, with microtubules appearing normal (Fig. 4A). Treatment with 20 µM nocodazole for 30 min resulted in a loss of organizing microtubule but left F-actin intact. These data confirm the specific effects of cytochalasin D and nocodazole on depolymerizing F-actin and microtubules, respectively. Treatment with cytochalasin D impaired the ability of α_1A_-AR to activate ERK1/2 but that with nocodazole did not affect the ERK1/2 activation at 20 min of PE stimulation and even increased the phosphorylation levels of ERK1/2 at a later stage of stimulation (Fig. 4B). However, activation of p38 seemed insensitive to the disruption of F-actin or microbules.

To further justify the role of endocytic trafficking in the ERK1/2 activation, we overexpressed amphiphysin, found to be critical for actin polymerization [29], in HEK-293A–α_1A_-AR cells. Overexpressed amphiphysin increased the phosphorylation level of ERK1/2 at 20- but not 50-min stimulation (Fig. 4C), which agrees with results showing that F-actin preferentially mediates the early-stage but later-stage trafficking of α_1A_-AR (Fig. 1). These data identified the association of F-actin in the ERK1/2 activation with α_1A_-AR agonist stimulation.

### ERK1/2 activation by α_1A_-AR is independent of the G_q_/PLC/PKC pathway

**Figure 5:**
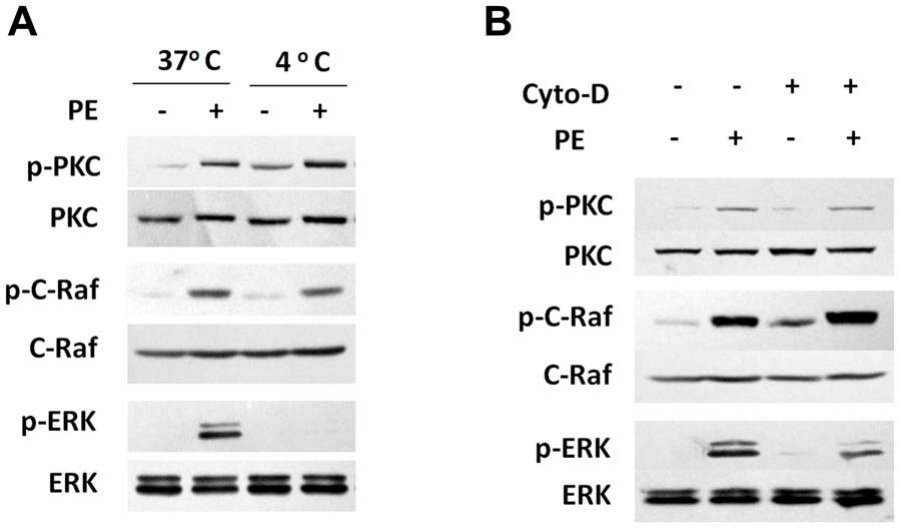
PKC and C-Raf activation with α_1A_-AR stimulation is not impaired by 4°C chilling or F-actin disruption. (A) HEK-293A–α_1A_-AR cells were pre-incubated at 4°C or 37°C for 30 min, then stimulated with 10 µM PE for 20 min. PKC, C-Raf and ERK1/2 activity was measured by western blot analysis. (B) HEK-293A–α_1A_-AR cells were pre-incubated with or without 5 µM cyto-D for 5 min, then stimulated with 10 µM PE for 20 min. PKC, C-Raf and ERK1/2 activity was measured by western blot analysis.

One of the earliest events in the signaling cascade initiated by α_1A_-AR is G_q_-mediated activation of PLC, with a resulting increase in PKC phosphorylation [14]. To test whether the G_q_/PLC/PKC pathway is involved in the α_1A_-AR-induced activation of ERK1/2, we examined the phosphorylation level of PKC and its downstream C-Raf molecule by α_1A_-AR with 4°C incubation or cytochalasin-D treatment. As compared with 37°C, 4°C incubation had no effect on the α_1A_-AR-activation of PKC or C-Raf, but it inhibited the activation of ERK1/2 (Fig. 5A). Similarly, pretreatment with cytochalasin-D before PE did not impair α_1A_-AR-induced PKC and C-Raf activation (Fig. 5B). Thus, α_1A_-AR endocytosis was not involved in the G_q_/PLC/PKC pathway.

**Figure 6:**
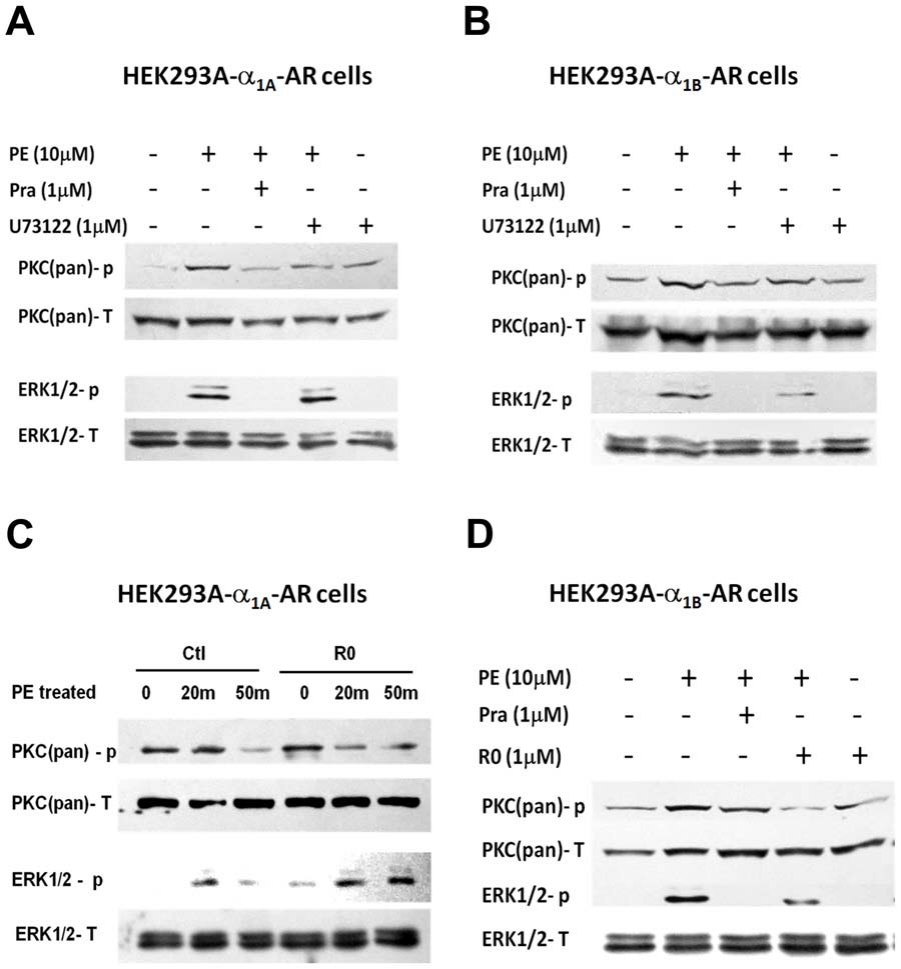
ERK1/2 activation by α_1A_-AR is independent of G_q_/PLC/PKC pathway. (A, B) Inhibition of phospholipase C (PLC) by U73122 affected ERK1/2 activation by α_1B_-AR but not α_1A_-AR. HEK-293A–α_1A_-AR or –α_1B_-AR cells were pre-incubated with 1 µM U73122 or prazosin or not for 30 min. Then cells were treated with 10 µM PE for 20 min. PKC and ERK1/2 activity was measured by western blot analysis. (C, D) Inhibition of PKC by Ro 31–8220 affected ERK1/2 activation by α_1B_-AR but not α_1A_-AR. HEK-293A–α_1A_-AR or –α_1B_-AR cells were pre-incubated with 1 µM Ro 31–8220 or prazosin or not for 30 min, then were treated with 10 µM PE for 20 min. PKC and ERK1/2 activity were measured by western blot analysis.

To detemine the role of G_q_/PLC/PKC signaling pathway in α_1A_-AR-induced ERK1/2 activation, HEK-293A–α_1A_-AR cells were treated with U73122 before PE exposure (Fig. 6A). U73122 is a pharmacological agent commonly used to demonstrate a role of G_q_ activation of PLC, which inhibits the PLC-dependent process [30]. U73122 suppressed PKC phosphorylation but did not alter the activation of ERK1/2 with PE stimulation. To compare the role of G_q_/PLC/PKC signaling in the activation of ERK1/2 by α_1A_-AR and α_1B_-AR, we also examined the effect of U73122 in HEK-293A cells stably transfected with α_1B_-AR. U73122 inhibited both PKC phosphorylation and ERK1/2 activation in HEK-293A–α_1B_-AR cells with PE stimulation (Fig. 6B). The PKC inhibitor Ro 31–8220 used before PE stimulation showed inhibition of PKC but not ERK1/2 activation with α_1A_-AR induction (Fig. 6C), and inhibition of both PKC and ERK1/2 with α_1B_-AR induction (Fig. 6D). The effects of U73122 and Ro 31–8220 on HEK-293A–α_1A_-AR or –α_1B_-AR cells suggest that the G_q_/PLC/PKC pathway is required for ERK1/2 activation induced by α_1B_-AR but not α_1A_-AR.

## Discussion

GPCRs activate MAPKS through distinct pathways in cells, and endosomal signaling has an important role in receptor signal transduction. We investigated the involvement of endocytosis in α_1A_-AR-induced activation of ERK1/2 in HEK-293A cells. Agonist-mediated endocytic trafficking of α_1A_-AR was assessed by real-time imaging of living, stably transfected cells. α_1A_-AR was internalized dynamically in cells with agonist stimulation, and actin filaments regulated the initial trafficking of α_1A_-AR. α_1A_-AR-induced activation of ERK1/2 but not p38 MAPK was sensitive to disruption of endocytosis. α_1A_-AR-induced activation of PKC and C-Raf by was not affected by endocytosis disruption. Thus, the endocytic pathway is involved in α_1A_-AR-induced ERK1/2 activation and is independent of G_q_/PLC/PKC signaling.

Real-time microscopy with high-tempo resolution has novel implications in the investigation of the behavior of receptors. For single particle tracking in this study, α_1A_-AR was detected on the surface of living HEK-293A–α_1A_-AR cells by use of a monoclonal primary antibody and Alexa-555 IgG. It should be aware of the potential effects of this labeling method on receptor trafficking and signaling. We found that the labeling with antibodies did not impair the calcium response of α_1A_-AR among the labeled cells, as compared with non-labeled ones [31]. And it did not affect the ERK1/2 signaling (See Supporting Information Figure S1). Previously, we showed α_1A_-AR transport with an average step size of 33 nm [21]. In the present work, α_1A_-AR showed a spatial and time-dependent trafficking pattern with PE stimulation. Near the plasma membrane, the α_1A_-AR particles were transported a relatively short distance (as shown in Fig. 1A and 1B, trajectory 1) within 8 sec. Inside the cells, the α_1A_-AR particles moved farther (as shown in Fig.1A and 1B, trajectory 2) in the same time interval. Long-distance transportation allows α_1A_-AR-containing vesicles to reach late endosomes or lysosomes, which were mostly located near the nucleus. At the early stage of the activation, the receptor mainly moved at a slow velocity, at a peak of about 0.3 µm/sec, which is consistent with the velocity of a single myosin motor walking along actin *in vitro* [32], [33]. After 40- to 60-min stimulation, the main peak of the higher velocity was about 0.8 µm/sec, which is similar to the value reported for movement along microtubules under *in vitro* conditions [34], [35]. Thus, the movement of α_1A_-AR vesicles mainly depends on actin at the early phase of endocytosis and on microtubules at the later phase. Confocal microscopy further verified that endocytic traffic of α_1A_-AR was mediated by reorganized actin (Fig. 2A, 2B).

Disruption of endocytosis by 4°C chilling, dynamin mutation and F-actin depolymerizing all indicated that the endocytic process was associated in α_1A_-AR-induced activation of ERK1/2. Many studies have implied a critical role for the ERK1/2 signaling pathway in cardiac myocytes [9]–[11]. Recently, α_1A_-AR was shown to signal through ERK1/2 to promote cardiac hypertrophy [36] or survival signals [12]. α_1A_-AR but not α_1B_-AR rescued α1ABKO cardiac myocytes from cell death, and only α_1A_-AR could mediate ERK1/2 activation in the myocytes [12]. Activation of phosphoinositide-3-kinase (PI3K) and Ras protein induces activation of a series of growth or proliferation-related protein kinase cascades [37]. Investigation of the role of PI3K and Ras in the activation of ERK1/2 by 2 α_1_-ARs in NIH3T3 cells revealed that overexpression of a dominant-negative Ras mutant attenuated the α_1B_-AR– but not α_1A_-AR-mediated activation of ERK1/2. And overexpression of a dominant-negative PI3K mutant (p85 subunit) attenuated α_1A_-AR- but not α_1B_-AR-induced ERK1/2 activation [38]. Therefore, α_1A_-AR and α_1B_-AR differentially activate downstream effectors, which further underscores the complexity of α_1_-AR signaling pathways. We found endocytic trafficking involved in α_1A_-AR induced ERK1/2 activation and that it was independent of G_q_/PLC/PKC signaling. By contrast, α_1B_-AR-induced ERK1/2 activation required G_q_/PLC/PKC signaling, as was previously reported [39]. Thus, α_1A_-AR uses pathways different from those of α_1B_-AR to activate ERK1/2.

A number of studies have found a non-G_q_-protein signaling pathway in α_1A_-AR function. An α_1A_-AR mutant defective in G_q_ coupling could also activate calcium influx when coactivated with β_2_-AR [16]. Thus, the functional interaction of these 2 receptors involves heterodimer formation and/or the presence of unidentified non-G_q_ signaling events in response to α_1A_-AR stimulation. Activation of calcium influx and PKC may be key events coupling G_q_- and G_i_-coupled receptor activation to MAPK activation [40]. Berts et al. showed that the Ca^2+^ chelator BAPTA dose-dependently abolished norepinephrine (NE) -stimulated Ca^2+^ responses but not ERK1/2 activation. The potent PKC inhibitor bisindolylmaleimide I dose dependently inhibited ERK1/2 activation by phorbol ester tumor-promoting agent but not NE. Thus, Ca^2+^ release and PKC activation are neither necessary nor sufficient for α_1A_-AR-mediated activation of ERK1/2 in PC12 cells stably transfected with α_1A_-AR [41]. Therefore, ERK1/2 activation induced by α_1A_-AR may be independent of traditional G_q_-coupled second-messenger pathways. We found that α_1A_-AR-induced ERK1/2 activation was unaffected by the PLC inhibitor U73122 and PKC inhibitor R0 31–822, which further identifies a non-G_q_ signaling pathway involved in α_1A_-AR-mediated ERK1/2 activation.

Arrestins are critically important for desensitization, endocytosis, and G protein-independent signaling of GPCRs [42]. One of the early evidences that β-arrestins are active participants in signaling was the observation that dominant-negative mutants of β-arrestins inhibited β_2_-AR-induced activation of ERK1/2 [18]. β-arrestins similarly participate in ERK1/2 signaling by other GPCRs, including neurokinin-1 receptor, protease activated receptor 2, angiotensin II type 1A receptor, and vasopressin V2 receptor [43]–[46]. The stability of receptor–β-arrestin complex controlled the mechanism and extent of ERK1/2 activation [44]. However, results from both coimmunoprecipitation experiments and β-arrestin translocation assays indicated that the agonist-induced interaction of α_1A_-AR with β-arrestins was much weaker than that of α_1B_-AR. In addition, α_1A_-AR did not bind AP50, a subunit of the clathrin adaptor complex AP2. Moreover epinephrine-induced increase of the association of the α_1A_-AR and β-arrestin 1 or 2 was not statistically significant [47]. Thus, defining the role of β-arrestins in the endocytosis of α_1A_-AR and α_1A_-AR-induced ERK1/2 activation is important. PI3K has a potential role in the α_1A_-AR induced ERK1/2 activation [38] and has been found to regulate intracellular vesicular transport at multiple steps [48], [49]. PI3K may be involved in α_1A_-AR mediated ERK1/2 activation through an endocytosis pathway but remains to be elucidated.

In summary, we identified a putative role of endocytosis in the regulation of the MAPK pathway under α_1A_-AR stimulation in cells. Through analysis of receptor tracking in live cells and confocal microscopy, we conclude that the α_1A_-AR endocytic process is actin-related. Endocytosis and actin reorganization are involved in α_1A_-AR induced activation of ERK1/2 but not p38, and G_q_/PLC/PKC signaling is not required in this process. Thus, we reveal a novel pathway of ERK1/2 activation in α_1_-AR subtypes. We provide the first biological evidence for a role of endocytosis in the signaling of the α_1_-AR family, which challenges the classical view that α_1A_-AR act through G_q_/PLC/PKC signaling. Moreover, the mechanism by which α_1A_-AR induces ERK1/2 activation differs from that of α_1B_-AR. We provide a possible molecular explanation for the difference between α_1A_-AR and α_1B_-AR in activation of ERK1/2 in cardiac myocytes. Because receptor vesicles could link to various intracellular membrane compartments in the endocytic process, the distinct spatiotemporal profile of ERK1/2 activation induced by α_1A_-AR has profound implications in α_1A_-AR-mediated survival signaling in cardiac myocytes. In-depth studies of this signaling pathway should add great potential for developing more efficacious and/or safer treatment of heart failure and other clinical conditions.

## Supporting Information

Figure S1 Labeling with antibodies did not impair the ERK1/2 signaling induced by α_1A_-AR stimulation. HEK-293A–α_1A_-AR cells were pre-incubated with or without anti-FLAG antibody and Alexa-555 IgG. After phenylephrine (PE) treatment, the phosphorylation level of ERK1/2 did not show significant difference among labeled and unlabeled cells. Prazosin (Pra, antagonist of α_1A_-AR) inhibited the PE-induced ERK1/2 activation in both.
(TIF)
