# Supplementary figures and images for: α1A-Adrenergic Receptor Induces Activation of Extracellular Signal-Regulated Kinase 1/2 through Endocytic Pathway
Source: PLoS One. 2011 Jun 28;6(6):e21520. doi: 10.1371/journal.pone.0021520 (PMC3125289; doi:10.1371/journal.pone.0021520)

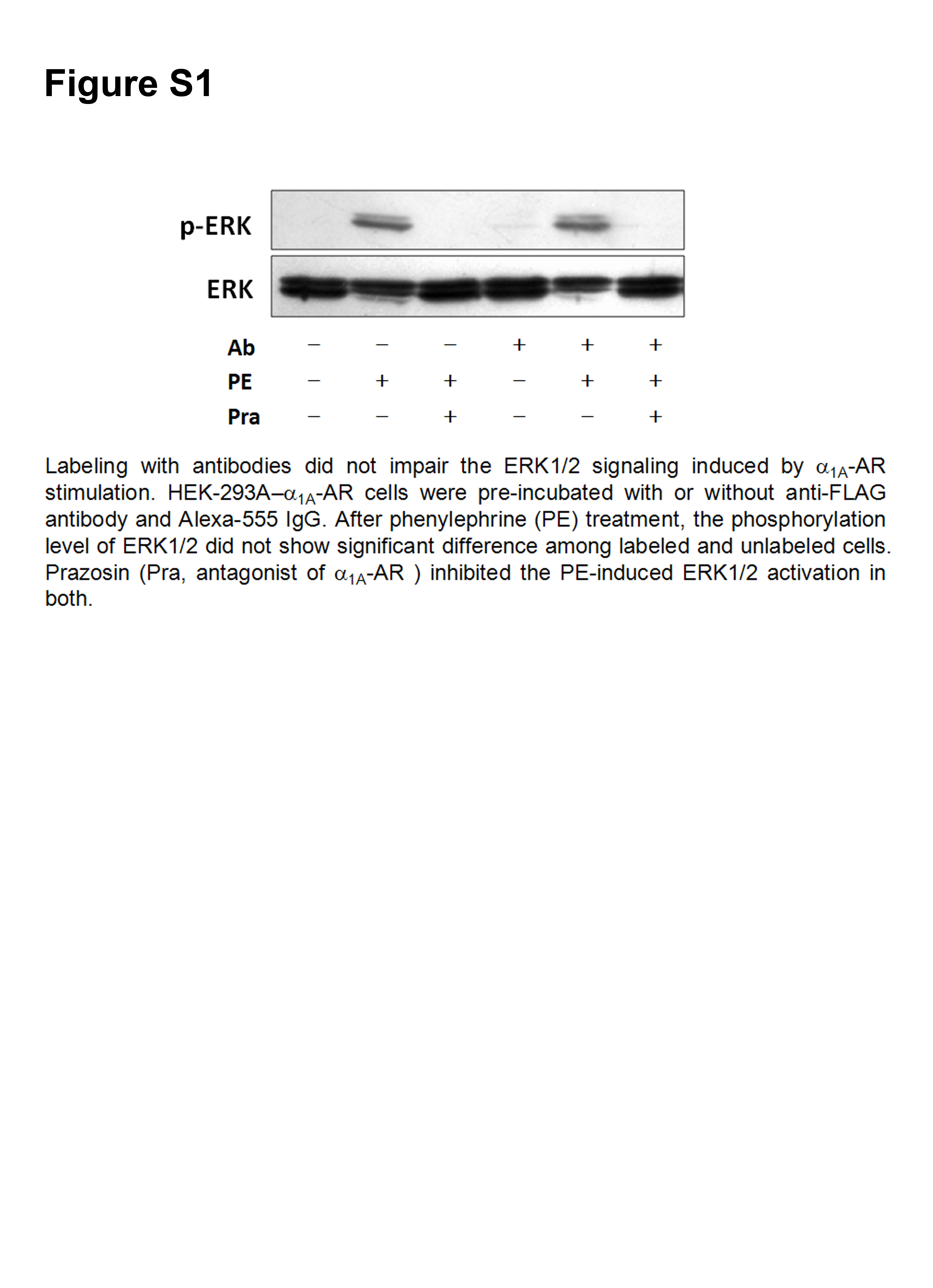

Supplement: Figure S1 — Labeling with antibodies did not impair the ERK1/2 signaling induced by α1A-AR stimulation. HEK-293A–α1A-AR cells were pre-incubated with or without anti-FLAG antibody and Alexa-555 IgG. After phenylephrine (PE) treatment, the phosphorylation level of ERK1/2 did not show significant difference among labeled and unlabeled cells. Prazosin (Pra, antagonist of α1A-AR) inhibited the PE-induced ERK1/2 activation in both. (TIF) [file pone.0021520.s001.tif]
